# Supplementary material for: Superconductivity in a van der Waals layered quasicrystal
Source: Nat Commun. 2024 Mar 1;15:1529. doi: 10.1038/s41467-024-45952-2 (PMC10907369; doi:10.1038/s41467-024-45952-2)
Supplement: Supplementary file 1 — Supplementary Information [file 41467_2024_45952_MOESM1_ESM.pdf]

Supplementary Information

Supplementary Discussion 1. Analyses of the powder X-ray diffractometry profiles

1.1. XRD peak indexing

The experimentally acquired XRD profile ($\mathrm{Cu\ K\alpha_1}$; $\lambda = 1.5405$ Å) was compared with those of the crystalline phases of the $TaTe_2$ and Ta materials employed in the reaction sintering (Supplementary Fig. 1). No traces of these phases were observed in the collected profile, indicating the completion of the reaction and formation of another phase.

An attempt was made to index the diffraction peaks in the acquired profile for the dodecagonal quasicrystal (QC). The electron diffraction pattern obtained from the sample revealed dodecagonal symmetry (Supplementary Fig. 2a). All the spots in the pattern were indexed using the four basis vectors $\mathbf{a}_i^*$ (i = 1–4) shown in the figure, with $a^* = |\mathbf{a}_i^*| \approx 0.69$ Å^{-1}. The dodecagonal QC phase reportedly exhibits the periodic stacking of a two-dimensional (2D) quasicrystalline layer with a period of $c \approx 10.4$ Å (ref. 1). This yields $c^* = |\mathbf{c}^*| = \frac{2\pi}{c} \approx 0.604$ Å^{-1}, where the basis vector $\mathbf{c}^*$ is perpendicular to $\mathbf{a}_i^*$. Using the five basis vectors $\mathbf{a}_i^*$ (i = 1–4) and $\mathbf{c}^*$, the peaks in the acquired XRD profile were successfully indexed, as shown in Supplementary Fig. 2b and Supplementary Table 1, with the values of a^* and c^* refined to 0.6942 and 0.6047 Å^{-1}, respectively.

1.2. Construction of the XRD profiles

In this section, the calculations performed to construct the profile of the dodecagonal QC phase using one of its crystal approximant (CA) phases are detailed, and a comparison between the calculated and acquired profiles is presented.

1.2.1. CA phases

To date, the structures of two Ta–Te CA phases with compositions $Ta_{21}Te_{13}$ and $Ta_{97}Te_{60}$ have been determined using single-crystal XRD measurements with the aid of

electron diffraction and high-resolution electron microscopy[2,3]. The projections of their structures onto the *c*-plane (Supplementary Figs. 3a and b) show atomic clusters with dodecagonal symmetry that are arranged periodically. Between the CA phases $Ta_{21}Te_{13}$ and $Ta_{97}Te_{60}$, the CA phase of $Ta_{97}Te_{60}$ was selected to calculate the dodecagonal QC phase profile. This is an orthorhombic (pseudo-tetragonal) crystal with lattice parameters a = 27.672, b = 27.672, and c = 20.613 Å, with the unit cell in the *ab*-plane indicated by the blue square in Supplementary Fig. 3b. The basic structure of this phase is a tetragonal crystal with lattice parameters a = 19.567 and c = 10.307 Å, with the unit cell in the *ab*-plane indicated by the red square in Supplementary Fig. 3b. A weak superlattice order is introduced into the basic structure of the $Ta_{97}Te_{60}$ phase. The XRD profiles calculated using VESTA software[4] from the determined structures of the two CA phases[2,3] were compared with the acquired profile (Supplementary Fig. 4). The overall features of the collected profile resembled those calculated for the CA phases, highlighting the similarity between the dodecagonal QC and CA phase structures. A more detailed comparison is presented in Supplementary Fig. 9.

1.2.2. Principles underlying the calculation

In general, by introducing phason strain to a QC structure, a series of periodic structures can be obtained, corresponding to the CA structures of the QC one. A typical one-dimensional (1D) quasiperiodic structure, called the Fibonacci lattice, which is expressed as a 1D section of a 2D periodic structure is shown in Supplementary Fig. 5a. Here, $E_{||}$ is the physical space, and $E_{\perp}$ is the complementary space perpendicular to $E_{||}$. Furthermore, a line segment on the 2D square lattice spanned by $\mathbf{A}_1$ and $\mathbf{A}_2$ exhibits a periodic arrangement, and a 1D point sequence is formed on $E_{||}$ as a set of line segment intersections with $E_{||}$. The slope of $E_{||}$ with respect to the 2D lattice is irrational ($\tau = (1+\sqrt{5})/2$ in this case), which leads to the point sequence on $E_{||}$ being quasiperiodic instead of periodic.

A phason-strained Fibonacci lattice is shown in Supplementary Fig. 5b. Phason strain can be imposed onto the Fibonacci lattice by applying shear strain to the 2D structure, as indicated by the arrows on the left and right sides of Supplementary Fig. 5a.

Upon introducing a shear strain, the vector $\mathbf{t} = \mathbf{A}_1 + 2\mathbf{A}_2$ shown in Supplementary Fig. 5a descends toward $\mathrm{E}_{||}$, as shown in Supplementary Fig. 5b. The structure resting atop $\mathrm{E}_{||}$ in Supplementary Fig. 5b exhibits periodicity with a period corresponding to its length. This periodic structure can be regarded as a 2/1 CA to the Fibonacci lattice, because the irrational slope of τ is replaced with a rational slope of 2/1.

The Fourier transform of the Fibonacci lattice can be calculated, as shown schematically in Supplementary Fig. 5c, where $\mathrm{E}_{||}^*$ and $\mathrm{E}_{\perp}^*$ represent the physical reciprocal space and its complementary space, respectively. First, the Fourier transform of the 2D periodic structure shown in Supplementary Fig. 5a was calculated; this comprised δ-functions at the 2D reciprocal lattice points ($\mathbf{G} = \mathbf{G}_{||} + \mathbf{G}_{\perp}$; $\mathbf{G}_{||} \in \mathrm{E}_{||}^*$, $\mathbf{G}_{\perp} \in \mathrm{E}_{\perp}^*$) spanned by the basis vectors $\mathbf{A}_1^*$ and $\mathbf{A}_2^*$ shown in Supplementary Fig. 5c. Their intensities were determined through the Fourier transform of the line segment, which was in the form $f(\mathbf{G}_{\perp}) = a\sin(b|\mathbf{G}_{\perp}|)/(b|\mathbf{G}_{\perp}|)$ (a, b: constants) and independent of $\mathbf{G}_{||}$. This indicates that the reciprocal lattice points with a large $|\mathbf{G}_{\perp}|$ constantly exhibited small intensities. The Fourier transform $F(\mathbf{q})$ of the Fibonacci lattice could be expressed as the projection of the δ-functions at the 2D reciprocal lattice points onto $\mathrm{E}_{||}^*$, as shown at the bottom of Supplementary Fig. 5c, that is:

$$\boldsymbol{F}(\mathbf{q}) = \sum_{\mathbf{G}} \delta(\mathbf{q} - \mathbf{G}_{||}) \cdot \boldsymbol{f}(\mathbf{G}_{\perp}). \quad (\mathrm{S1})$$

Here, $\mathbf{G}_{||} = h_1\mathbf{a}_1^* + h_2\mathbf{a}_2^*$ ($h_1, h_2 \in$ integers), where $\mathbf{a}_1^*$ and $\mathbf{a}_2^*$ are the projections of $\mathbf{A}_1^*$ and $\mathbf{A}_2^*$ onto $\mathrm{E}_{||}^*$, respectively. Because $|f(\mathbf{G}_{\perp})|$ is constantly small for a large $|\mathbf{G}_{\perp}|$, a threshold value $G_{\perp}^0$ (> 0) could be used for $|\mathbf{G}_{\perp}|$, and the summation in equation (S1) for $\mathbf{G}$ could be limited to $|\mathbf{G}_{\perp}| < G_{\perp}^0$, that is:

$$F(\mathbf{q}) = \sum_{\mathbf{G}\text{ with }|\mathbf{G}_{\perp}|<G_{\perp}^0} \delta(\mathbf{q} - (h_1\mathbf{a}_1^* + h_2\mathbf{a}_2^*)) \cdot f(\mathbf{G}_{\perp}). \quad (S2)$$

The Fourier transform of the 2/1 CA was performed based on the fundamental Fourier transformation attributes, as shown in Supplementary Fig. 5d. This corresponds to the projection of the Fourier transform of the 2D structure shown in Supplementary Fig. 5b. The 2D Fourier transform shown in Supplementary Fig. 5d was obtained by introducing a shear strain to the 2D Fourier transform shown in Supplementary Fig. 5c,

as indicated by the arrows on the top and bottom of the figure, which led to the vector $\mathbf{t}^* = -2\mathbf{A}_1^* + \mathbf{A}_2^*$ descending toward $\mathrm{E}_\perp^*$. Finally, the Fourier transform of the 2/1 CA was obtained by projecting the 2D Fourier transform onto $\mathrm{E}_{||}^*$, which comprised δ-functions, as shown at the bottom of Supplementary Fig. 5d. If an appropriate value is adopted for $G_\perp^0$, no more than one 2D reciprocal lattice point will fall onto $\mathrm{E}_{||}^*$ at the same position. Consequently, the Fourier transform of the 2/1 CA can be expressed in the same form as that of equation (S2), but with a different set of basis vectors.

$$F(\mathbf{q}) = \sum_{\mathbf{G}\text{ with }|\mathbf{G}_\perp|<G_\perp^0} \delta\big(\mathbf{q} - (h_1\mathbf{b}_1^* + h_2\mathbf{b}_2^*)\big) \cdot f(\mathbf{G}_\perp), \qquad \text{(S3)}$$

where $\mathbf{b}_1^*$ and $\mathbf{b}_2^*$ are the projections of the basis vectors $\mathbf{B}_1^*$ and $\mathbf{B}_2^*$, respectively, in the 2D reciprocal lattice shown in Supplementary Fig. 5d. Here, the position of the δ-function, $h_1\mathbf{b}_1^* + h_2\mathbf{b}_2^*$, can be reindexed using only one basis vector $\mathbf{d}_1^* = \mathbf{b}_1^*$ as $H_1\mathbf{d}_1^*$, where $H_1 = h_1 + 2h_2$. Appropriate selection of $G_\perp^0$ assures one-to-one correspondence between (h_1, h_2) and H_1. Consequently, if the diffraction intensity function ($I(\mathbf{q}) \equiv |F(\mathbf{q})|^2$) for the Fibonacci lattice can be determined, that for the 2/1 CA can be obtained simply by shifting each δ-function from $h_1\mathbf{a}_1^* + h_2\mathbf{a}_2^*$ to $H_1\mathbf{d}_1^* = (h_1 + 2h_2)\mathbf{d}_1^*$ without changing the intensity and vice versa. This argument should generally hold true for a QC and its CA. Subsequently, this scheme was applied to the dodecagonal QC phase and one of its CA phases, and the XRD profile of the dodecagonal QC phase was calculated using that of its CA phase.

1.2.3. Four-dimensional description of a dodecagonal QC and its CAs[5]

For a dodecagonal QC and its CAs, a four-dimensional (4D) space spanned by orthonormal unit vectors $\mathbf{e}_i$ $(i = 1 - 4)$ can be considered, with $\mathbf{e}_1$ and $\mathbf{e}_2$ spanning the physical space $\mathrm{E}_{||}$ ($\mathrm{E}_{||}^*$) and $\mathbf{e}_3$ and $\mathbf{e}_4$ encompassing the complementary space $\mathrm{E}_\perp$ ($\mathrm{E}_\perp^*$); these can be compared with those in Supplementary Figs. 5a–d. A 4D dodecagonal lattice is spanned by $\mathbf{A}_i$ $(i = 1 - 4)$ expressed as:

$$\mathbf{A}_i = \sum_{j=1}^{4} M_{ij}\mathbf{e}_j,$$

$$M = \frac{a_{4\mathrm{D}}}{\sqrt{2}}\begin{pmatrix} \sqrt{3}/2 & -1/2 & -\sqrt{3}/2 & -1/2 \\ 1 & 0 & 1 & 0 \\ 0 & 1 & 0 & 1 \\ -1/2 & \sqrt{3}/2 & -1/2 & -\sqrt{3}/2 \end{pmatrix}. \quad \text{(S4)}$$

Here, a_{4D} is the lattice constant of the 4D lattice. The lattice reciprocal to this 4D lattice is spanned by $\mathbf{A}_i^*$ $(i = 1-4)$ and is expressed as:

$$\mathbf{A}_i^* = \sum_{j=1}^{4} {}^tM_{ij}^{-1}\mathbf{e}_j,$$

$${}^tM_{ij}^{-1} = \frac{a_{4\mathrm{D}}^*}{\sqrt{2}}\begin{pmatrix} 1 & 0 & -1 & 0 \\ \sqrt{3}/2 & 1/2 & \sqrt{3}/2 & -1/2 \\ 1/2 & \sqrt{3}/2 & -1/2 & \sqrt{3}/2 \\ 0 & 1 & 0 & -1 \end{pmatrix}. \quad \text{(S5)}$$

Here, the superscript t denotes transposition, and $a_{4\mathrm{D}}^*$ $(= 4\pi/(\sqrt{3}a_{4\mathrm{D}}))$ represents the lattice constant of the 4D reciprocal lattice. The projections of $\mathbf{A}_i$ $(\mathbf{A}_i^*)$ $(i = 1-4)$—$\mathbf{a}_i$ $(\mathbf{a}_i^*)$ and $\mathbf{u}_i$ $(\mathbf{u}_i^*)$—onto $\mathrm{E}_{||}$ $(\mathrm{E}_{||}^*)$ and $\mathrm{E}_{\perp}$ $(\mathrm{E}_{\perp}^*)$, respectively, is shown in Supplementary Fig. 6.

A series of square CAs can be obtained by imposing phason strain onto the 4D dodecagonal lattice, which causes the following two vectors $\mathbf{t}_1$ and $\mathbf{t}_2$ to descend toward $\mathrm{E}_{||}$ (see Supplementary Figs. 5a and b):

$$\begin{aligned} \mathbf{t}_1 &= (2p, q, p, 0)_{\mathbf{A}_i} \\ \mathbf{t}_2 &= (0, p, q, 2p)_{\mathbf{A}_i}, \end{aligned} \quad \text{(S6)}$$

where p and q are integers, and the subscript $\mathbf{A}_i$ indicates that the vectors are represented with respect to the basis vectors $\mathbf{A}_i$ $(i = 1-4)$. The vectors $\mathbf{t}_1$ and $\mathbf{t}_2$ can be rewritten using equation (S4) with the basis vectors $\mathbf{e}_i$ $(i = 1-4)$ as:

$$\begin{aligned} \mathbf{t}_1 &= \frac{a_{4\mathrm{D}}}{\sqrt{2}}(\sqrt{3}p + q, 0, -\sqrt{3}p + q, 0)_{\mathbf{e}_i} \\ \mathbf{t}_2 &= \frac{a_{4\mathrm{D}}}{\sqrt{2}}(0, \sqrt{3}p + q, 0, -\sqrt{3}p + q)_{\mathbf{e}_i} \end{aligned}, \quad \text{(S7)}$$

Equation (S7) indicates that (1) the $\mathrm{E}_{\perp}$ components of $\mathbf{t}_1$ and $\mathbf{t}_2$ approach zero as $q/p \rightarrow \sqrt{3}$; (2) the $\mathrm{E}_{||}$ components of $\mathbf{t}_1$ and $\mathbf{t}_2$ are oriented along $\mathbf{e}_1$ and $\mathbf{e}_2$, respectively, corresponding to the lattice translational vectors of the square CA structure; and (3) the lattice constant of the square lattice can be expressed as:

$$a = \frac{\sqrt{3}p + q}{\sqrt{2}} a_{4\mathrm{D}}. \quad \text{(S8)}$$

Point (1) suggests that the closer *q*/*p* is to $\sqrt{3}$, the more identical is the CA structure to the QC structure.

The introduction of the phason strain changes the basis vectors of the 4D dodecagonal lattice $\mathbf{A}_i$ $(i = 1 - 4)$ to $\mathbf{B}_i$ $(i = 1 - 4)$. Correspondingly, the basis vectors of the reciprocal lattice $\mathbf{A}_i^*$ $(i = 1 - 4)$ are transformed into $\mathbf{B}_i^*$ $(i = 1 - 4)$, similar to the case of the Fibonacci lattice shown in Supplementary Figs. 5a–d. On the other hand, equation (S7) and the relationship $a_{4\mathrm{D}}^* = 4\pi/(\sqrt{3}a_{4\mathrm{D}})$ indicate that the reciprocal basis vectors of the square lattice of the CA—$\mathbf{d}_1^*$ and $\mathbf{d}_2^*$—can be expressed with respect to the basis vectors $\mathbf{e}_1$ and $\mathbf{e}_2$ as:

$$\begin{aligned} \mathbf{d}_1^* &= \frac{a_{4\mathrm{D}}^*}{\sqrt{2}}\left(\frac{1}{p + q/\sqrt{3}}, 0\right) \\ \mathbf{d}_2^* &= \frac{a_{4\mathrm{D}}^*}{\sqrt{2}}\left(0, \frac{1}{p + q/\sqrt{3}}\right) \end{aligned}, \quad \text{(S9)}$$

Based on the relationship between the projections of $\mathbf{B}_i^*$ $(i = 1 - 4)$ onto $\mathrm{E}_{||}^*$ (that is, $\mathbf{b}_i^*$ $(i = 1 - 4)$) and $\mathbf{d}_1^*$ $(i = 1,2)$, the δ-functions at $h_1\mathbf{a}_1^* + h_2\mathbf{a}_2^* + h_3\mathbf{a}_3^* + h_4\mathbf{a}_4^*$ for the dodecagonal QC are linked to those at $H_1\mathbf{d}_1^* + H_2\mathbf{d}_2^*$ for the CA through the expression:

$$\begin{pmatrix} H_1 \\ H_2 \end{pmatrix} = \begin{pmatrix} 2p & q & p & 0 \\ 0 & p & q & 2p \end{pmatrix} \begin{pmatrix} h_1 \\ h_2 \\ h_3 \\ h_4 \end{pmatrix}. \quad \text{(S10)}$$

1.2.4. Parameter determination

a^* was determined to be 0.6942 Å^{-1} for the investigated dodecagonal QC phase (see Section 1.1). This value should be equal to $\frac{a_{4\mathrm{D}}^*}{\sqrt{2}}$ in equation (S5), which leads to $a_{4\mathrm{D}}^* = 0.9817$ Å^{-1}. Furthermore, the relationship $a_{4\mathrm{D}}^* = 4\pi/(\sqrt{3}a_{4\mathrm{D}})$ yields an a_{4D} value of 7.390 Å. The lattice constant (a) of the basic structure of the $Ta_{97}Te_{60}$ CA phase was determined to be 19.567 Å (Section 1.2.1)[3]. Notably, for *p* = 1 and *q* = 2, equation (S8)

yields a = 19.50 Å, which is consistent with the lattice constant of the CA phase. Therefore, the $Ta_{97}Te_{60}$ phase was confirmed to be a square CA phase with p = 1 and q = 2 (tetragonal in 3D). Considering the superlattice ordering in the *ab*-plane and along the *c*-direction in the CA phase, as described in Section 1.2.1, the indices (H_1', H_2', H_3') for the superlattice-ordered structure are related to those (H_1, H_2, H_3) for the basic structure as follows:

$$\begin{pmatrix} H_1' \\ H_2' \\ H_3' \end{pmatrix} = \begin{pmatrix} 1 & -1 & 0 \\ 1 & 1 & 0 \\ 0 & 0 & 2 \end{pmatrix} \begin{pmatrix} H_1 \\ H_2 \\ H_3 \end{pmatrix}. \quad (S11)$$

Using equations (S11) and (S10) with p = 1 and q = 2, the relationship between the indices (H_1', H_2', H_3') for the superlattice-ordered $Ta_{97}Te_{60}$ CA phase and those $(h_1, h_2, h_3, h_4, h_5)$ for the dodecagonal QC phase can be obtained.

$$\begin{pmatrix} H_1' \\ H_2' \\ H_3' \end{pmatrix} = \begin{pmatrix} 2 & 1 & -1 & -2 & 0 \\ 2 & 3 & 3 & 2 & 0 \\ 0 & 0 & 0 & 0 & 2 \end{pmatrix} \begin{pmatrix} h_1 \\ h_2 \\ h_3 \\ h_4 \\ h_5 \end{pmatrix} \quad (S12)$$

$G_\perp^0$ was assumed to be $2.4\frac{a_{4\mathrm{D}}^*}{\sqrt{2}}$, and the XRD profile of the dodecagonal QC phase was calculated using that of the $Ta_{97}Te_{60}$ CA phase with equation (S12).

1.2.5. Results

The indices, q-value, the structural factor squared $|F|^2$, scattering angle 2θ, and powder diffraction intensity I calculated for the $Ta_{97}Te_{60}$ CA phase using VESTA software[4] with the structural data reported by Conrad & Harbrecht[3], where $q = (4\pi \sin\theta)/\lambda$, and $\lambda = 1.5405$ Å^{-1} (Cu Kα_1) are listed in Supplementary Data 1. The calculation results of the dodecagonal QC phase are summarized in Supplementary Data 2. The diffraction patterns calculated for the $Ta_{97}Te_{60}$ CA and dodecagonal QC phases on the planes perpendicular to the *c*-axis, respectively, are shown in Supplementary Figs. 7a and b. The collected powder XRD profile of the sample and those calculated for the dodecagonal QC, $Ta_{97}Te_{60}$ CA, and $Ta_{21}Te_{13}$ CA phases are illustrated in

Supplementary Fig. 8; Supplementary Figs. 9a–d present the magnified versions of these profiles.

Supplementary Discussion 2. Debye temperatures

In Supplementary Fig. 10, the Debye temperatures (Θ_{D}) of various layered Ta-chalcogenides[6–10], including those in Table 1, are plotted against ${M_{\mathrm{ave}}}^{-1/2}$ (M_{ave}: average atomic weight). The relationship $\Theta_{\mathrm{D}} \propto {M_{\mathrm{ave}}}^{-1/2}$ approximately holds except for Ta_2Se, whose Θ_{D} is considerably high for unknown reasons.

References

1. Conrad, M., Krumeich, F. & Harbrecht, B. A dodecagonal quasicrystalline chalcogenide. *Angew. Chem. Int. Ed.* **37**, 1383–1386 (1998).
2. Conrad, M., Krumeich, F., Reich, C. & Harbrecht, B. Hexagonal approximants of a dodecagonal tantalum telluride – the crystal structure of $Ta_{21}Te_{13}$. *Mater. Sci. Eng. A* **294–296**, 37–40 (2000).
3. Conrad, M. & Harbrecht, B. $Ta_{97}Te_{60}$: A Crystalline Approximant of a Tantalum Telluride Quasicrystal with Twelvefold Rotational Symmetry. *Chem. Eur. J.* **8**, 3093–3102 (2002).
4. Momma, K. & Izumi, F. VESTA 3 for three-dimensional visualization of crystal, volumetric and morphology data. *J. Appl. Crystallogr.* **44**, 1272–1276 (2011).
5. Yamamoto, A. Crystallography of Quasiperiodic Crystals. *Acta Cryst.* **A52**, 509–560 (1996).
6. Gui, X., Górnicka, K., Chen, Q., Zhou, H., Klimczuk, T. & Xie, W. Superconductivity in metal-rich chalcogenide Ta_2Se. *Inorg. Chem.* **59**, 5798–5802 (2020).
7. Bhoi, D., Khim, S., Nam, W., Lee, B. S., Kim, C., Jeon, B.-G., Min, B. H., Park, S. & Kim, K. H. Interplay of charge density wave and multiband superconductivity in 2*H*-Pd_xTaSe_2. *Sci. Rep.* **6**, 24068 (2016).
8. Luo, H., Xie, W., Tao, J., Inoue, H., Gyenis, A., Krizan, J. W., Yazdani, A., Zhu, Y. & Cava, R. J. Polytypism, polymorphism, and superconductivity in $TaSe_{2-x}Te_x$. *Proc. Natl. Acad. Sci. U.S.A.* **112**, E1174–E1180 (2015).
9. Wagner, K. E., Morosan, E., Hor, Y. S., Tao, J., Zhu, Y., Sanders, T., McQueen, T. M., Zandbergen, H. W., Williams, A. J., West, D. V. & Cava, R. J. Tuning the charge density wave and superconductivity in Cu_xTaS_2. *Phys. Rev. B* **78**, 104520 (2008).

10. Ribak, A., Skiff, R. M., Mograbi, M., Rout, P. K., Fischer, M. H., Ruhman, J., Chashka, K., Dagan, Y. & Kanigel, A. Chiral superconductivity in the alternate stacking compound 4Hb-TaS_2. *Sci. Adv.* **6**, eaax9480 (2020).

Supplementary Table 1 | XRD peak indexing results. q_{exp} and q_{calc} denote the experimental and calculated q-values, respectively. $q = \frac{4\pi \sin\theta}{\lambda}$, and $\lambda = 1.5405$ Å.

q_{exp}	Index	q_{calc}
0.608	00001	0.6047
0.688	$1\bar{1}001$	0.7034
0.912	10001	0.9206
1.220	00002	1.2094
1.340	11000	1.3411
1.901	11100	1.8966
1.991	11101	1.9906
2.596	12100	2.5908
2.665	12101	2.6604

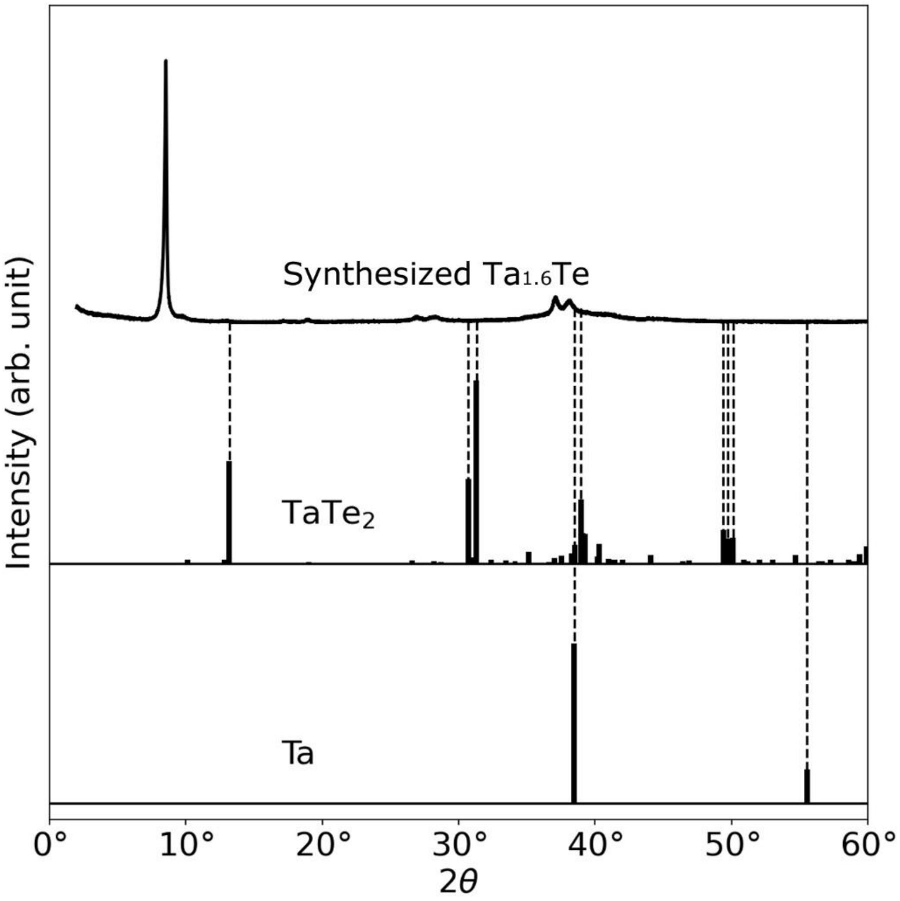

Supplementary Fig. 1 | Powder XRD analysis. Comparison between the experimentally acquired powder XRD profile of the $Ta_{1.6}Te$ sample ($\mathrm{Cu\ K\alpha_1}$; $\lambda = 1.5405$ Å) and the peak data of the crystalline phases of $TaTe_2$ and Ta obtained from 'Powder Diffraction Datafile (PDF)'.

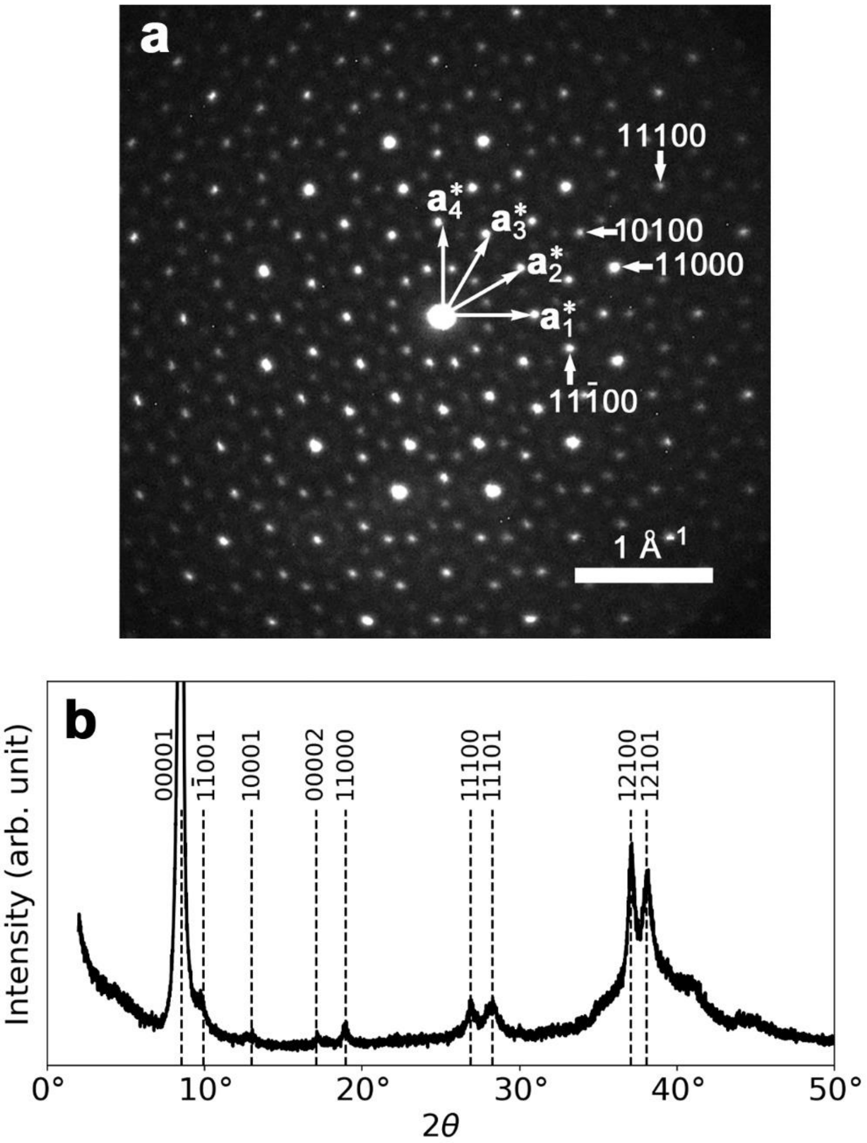

Supplementary Fig. 2 | XRD peak indexing. **a**, A twelve-fold electron diffraction pattern showing the four basis vectors $\mathbf{a}_i^*$ (i = 1–4) used for indexing. **b**, Results of the XRD peak indexing.

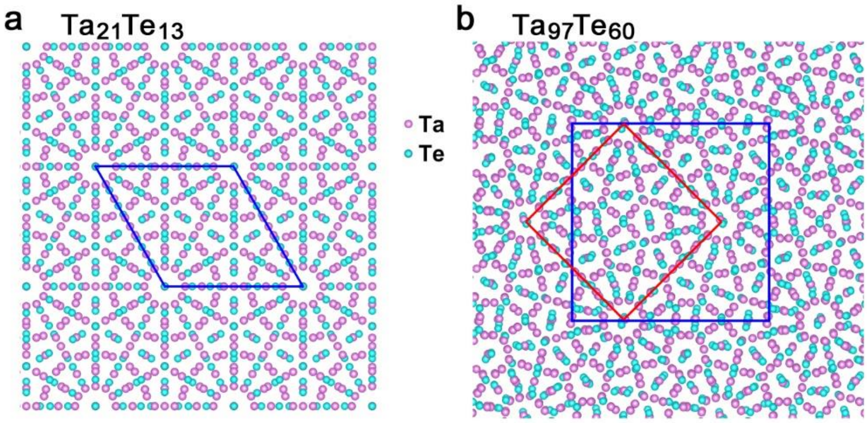

Supplementary Fig. 3 | Structures of the CA phases. a, b, Projections of the $Ta_{21}Te_{13}$ (**a**) and $Ta_{97}Te_{60}$ (**b**) CA phase structures onto the *c*-plane[2,3]. The unit cells of the crystals are indicated in blue, and that of the basic structure in **b** is shown in red.

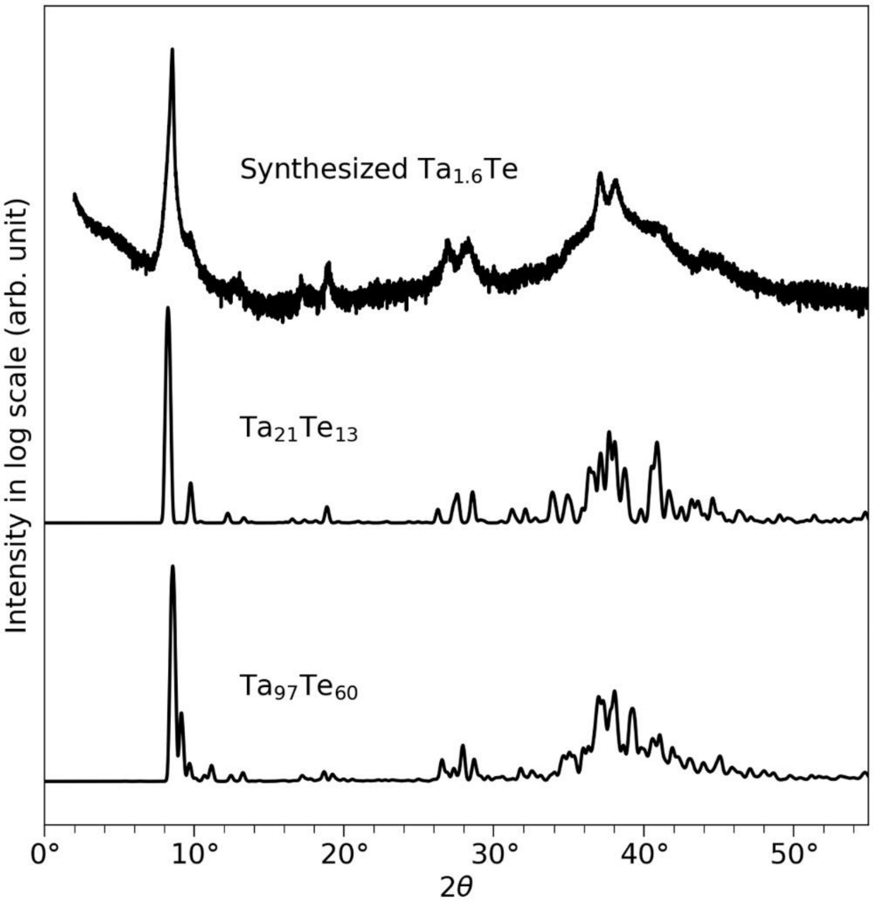

Supplementary Fig. 4 | Powder XRD investigation. Experimentally obtained powder XRD profile of the $Ta_{1.6}Te$ sample ($\mathrm{Cu}\ \mathrm{K}\alpha_1$; $\lambda = 1.5405$ Å) and calculated profiles of the $Ta_{21}Te_{13}$ and $Ta_{97}Te_{60}$ CA phases. Gaussians with a full width at half maximum of $\Delta q = 0.02$ Å^{-1} were used for the calculated profiles. Intensities are shown on a logarithmic scale so that weak spots are clearly visible.

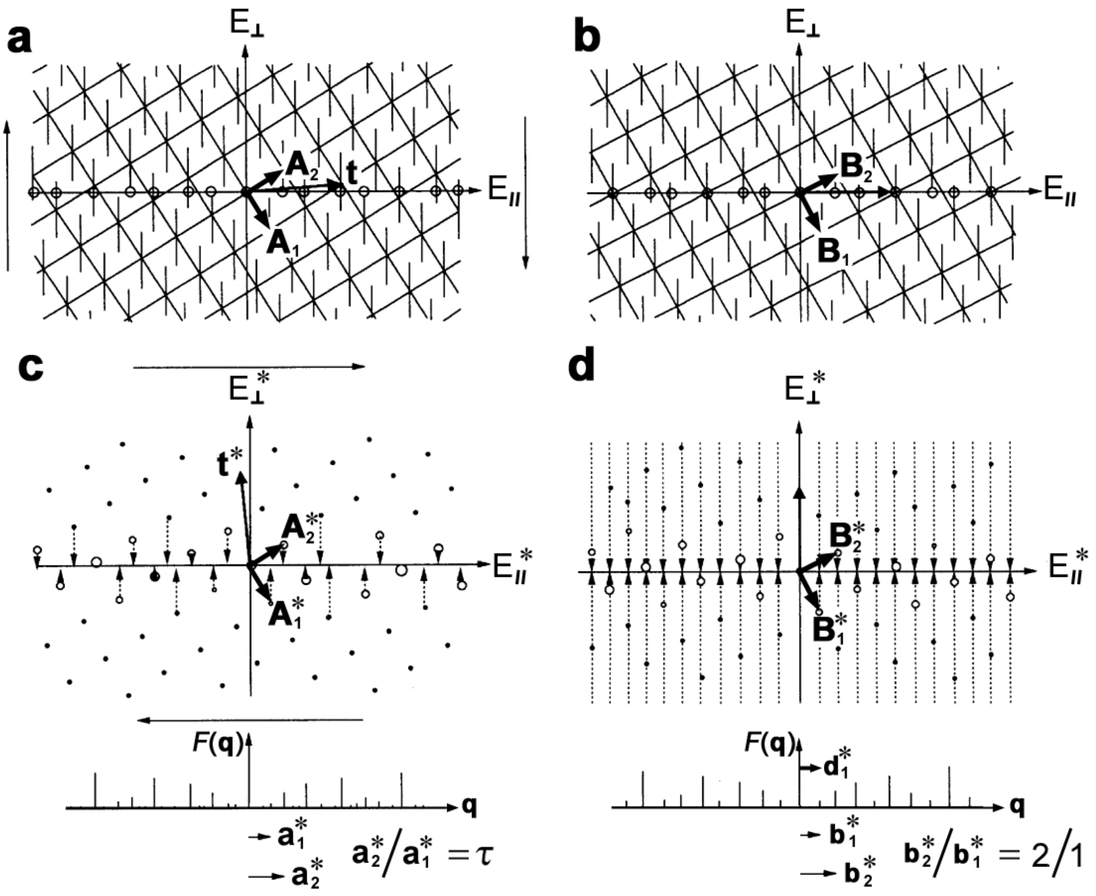

Supplementary Fig. 5 | Relationships between a QC and its CA in real and reciprocal spaces a, A 1D quasiperiodic structure, called a Fibonacci lattice, expressed as a 1D section of a 2D periodic structure. **b**, A CA obtained upon imposing a phason strain onto the Fibonacci lattice shown in **a**. **c**, **d**, Fourier transforms of the Fibonacci lattice (**c**) and CA (**d**).

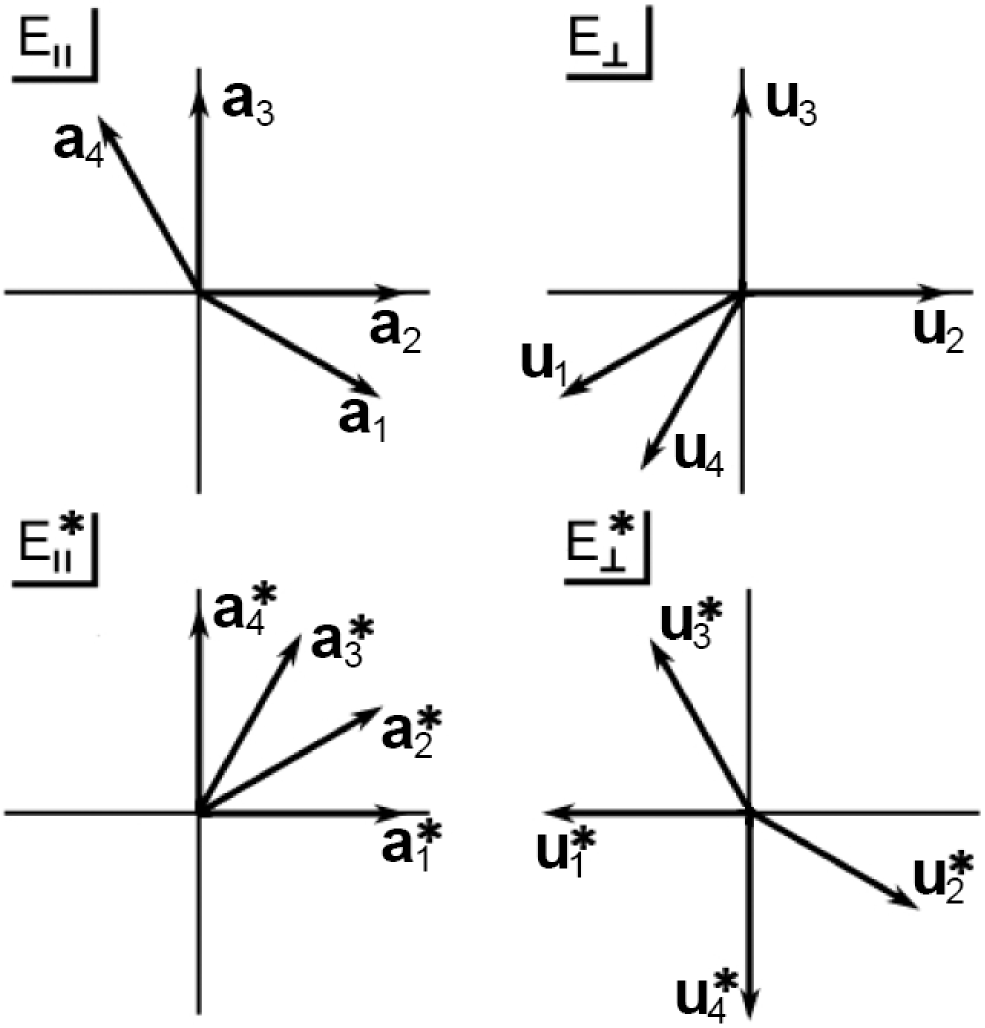

Supplementary Fig. 6 | Projections of the basis vectors of a 4D dodecagonal lattice. Projections of the basis vectors $\mathbf{A}_i$ ($\mathbf{A}_i^*$) ($i = 1 - 4$) of a 4D dodecagonal lattice (that is, $\mathbf{a}_i$ ($\mathbf{a}_i^*$) and $\mathbf{u}_i$ ($\mathbf{u}_i^*$)) onto $E_{||}$ ($E_{||}^*$) and $E_{\perp}$ ($E_{\perp}^*$), respectively.

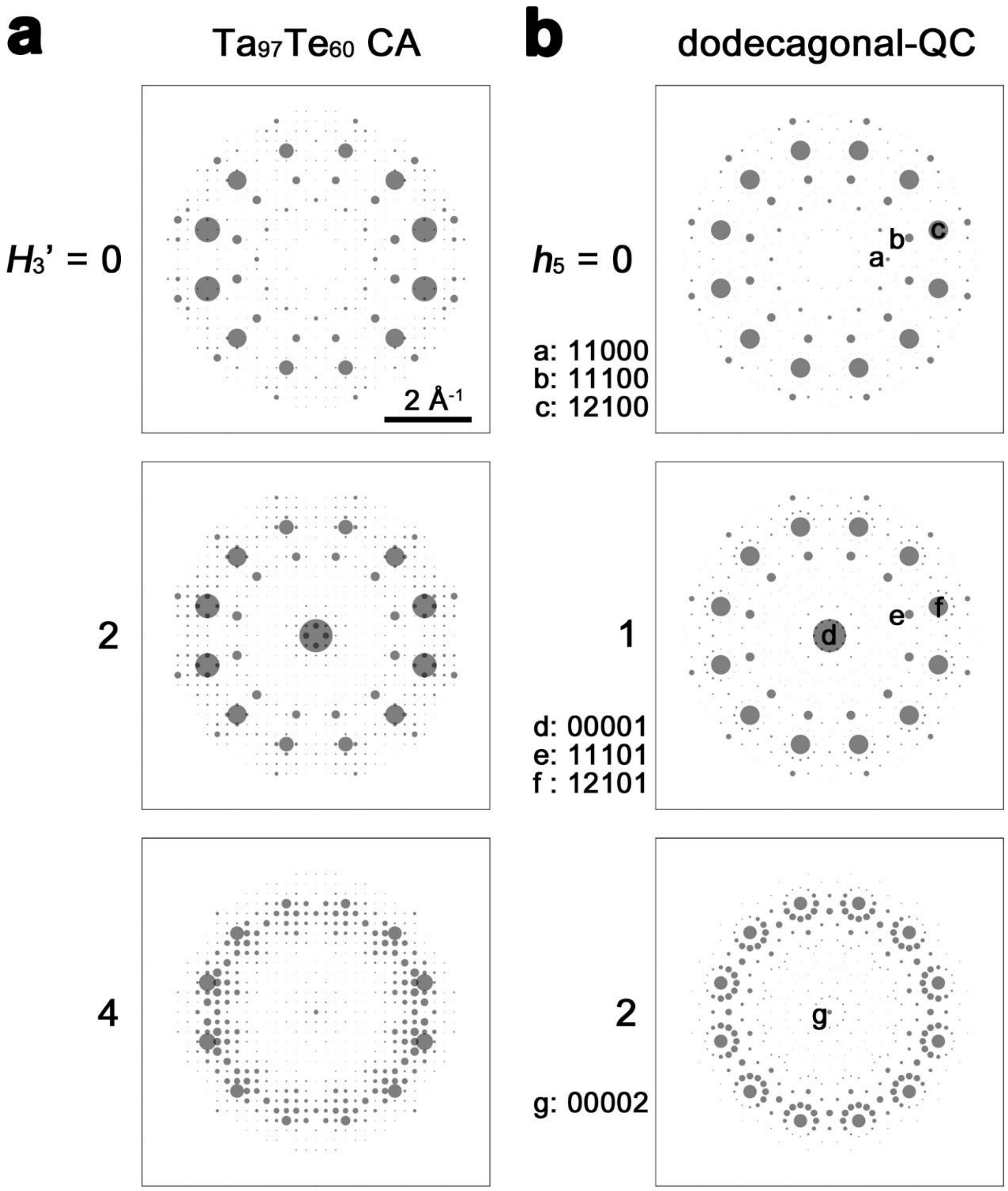

Supplementary Fig. 7 | Diffraction patterns of the CA and dodecagonal QC phases. **a, b**, Calculated diffraction patterns of the $Ta_{97}Te_{60}$ CA (**a**) and dodecagonal QC (**b**) phases on the planes perpendicular to the *c*-axis. For the dodecagonal QC phase, averaging has been made for symmetry-related diffraction spots. The area of the circle is proportional to $|F|^2$.

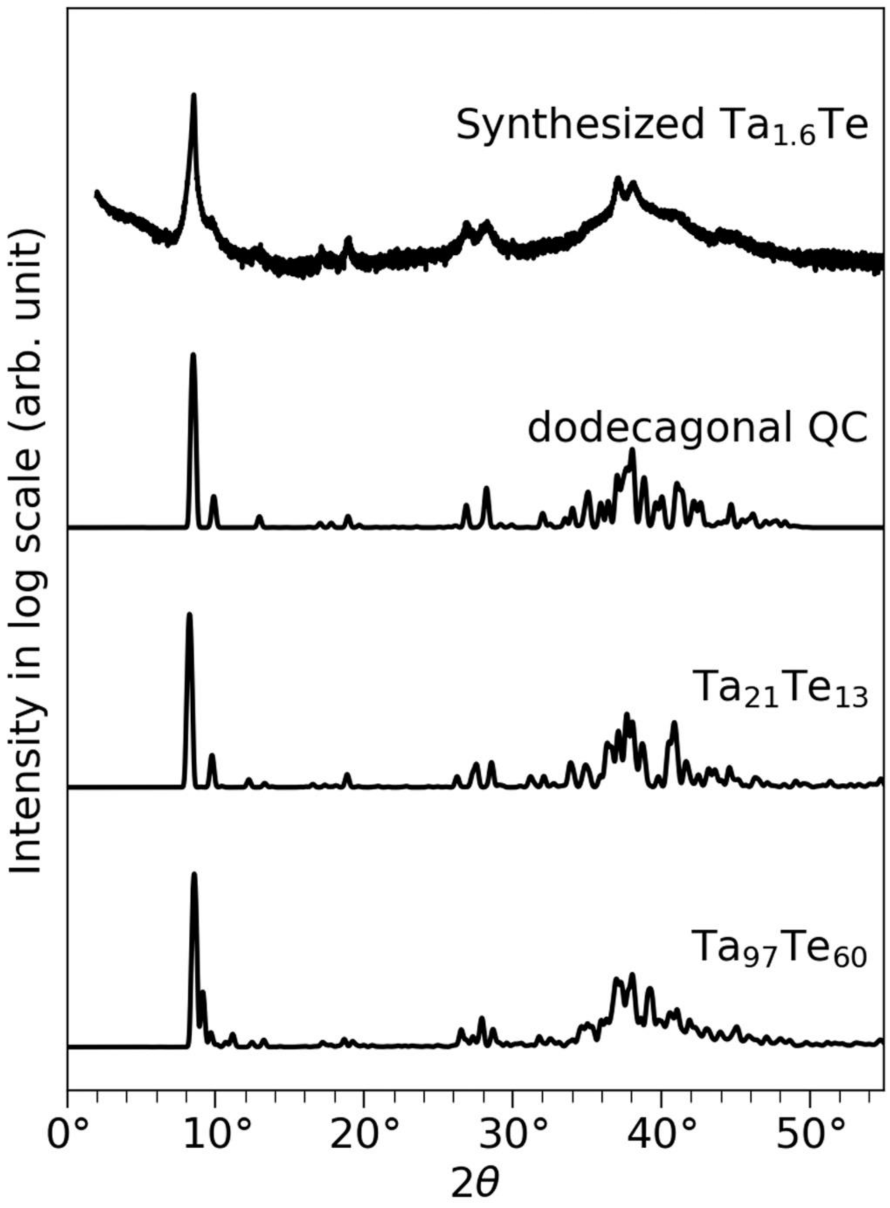

Supplementary Fig. 8 | Powder XRD analysis. Powder XRD profiles acquired for the synthesized $Ta_{1.6}Te$ sample and calculated for the dodecagonal QC, $Ta_{21}Te_{13}$ CA, and $Ta_{97}Te_{60}$ CA phases. Gaussians with a full width at half maximum of $\Delta q = 0.02$ Å^{-1} were used for the calculated profiles. Intensities are shown on a logarithmic scale so that weak spots are clearly visible.

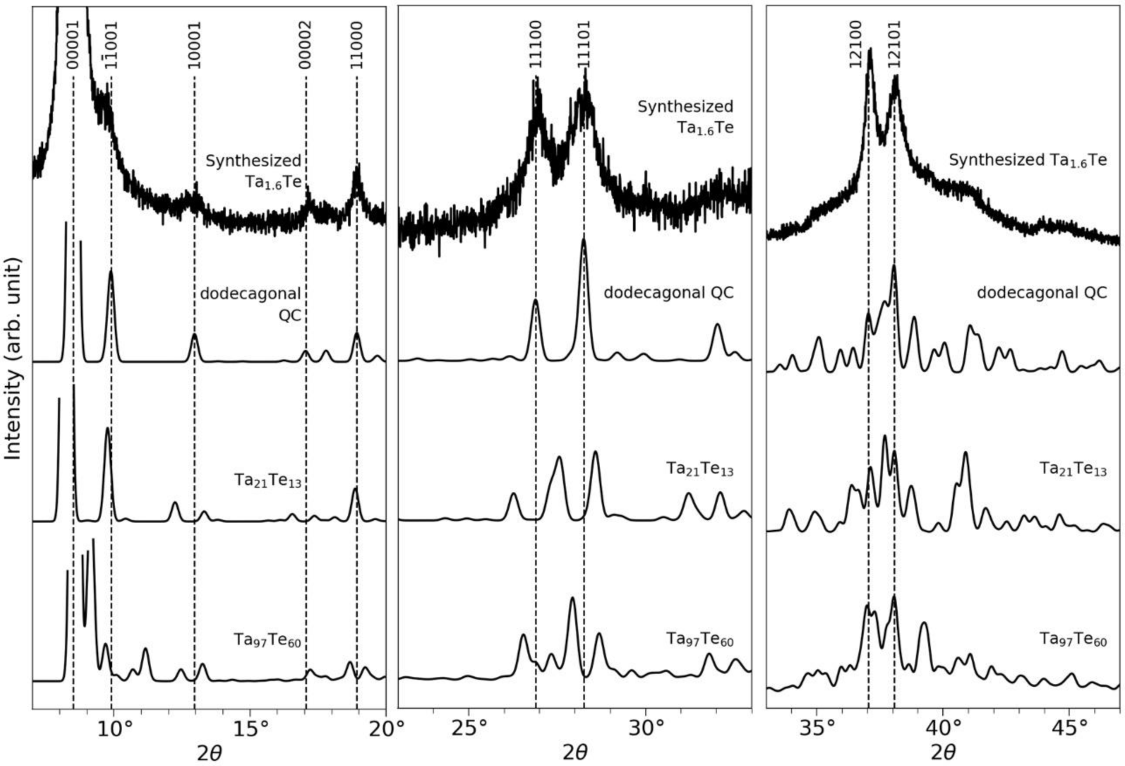

Supplementary Fig. 9 | Magnified versions of the powder XRD profiles shown in Supplementary Fig. 8. Powder XRD profiles acquired for the synthesized $Ta_{1.6}Te$ sample and calculated for the dodecagonal QC, $Ta_{21}Te_{13}$ CA, and $Ta_{97}Te_{60}$ CA phases. Gaussians with a full width at half maximum of $\Delta q = 0.02$ Å^{-1} were used for the calculated profiles.

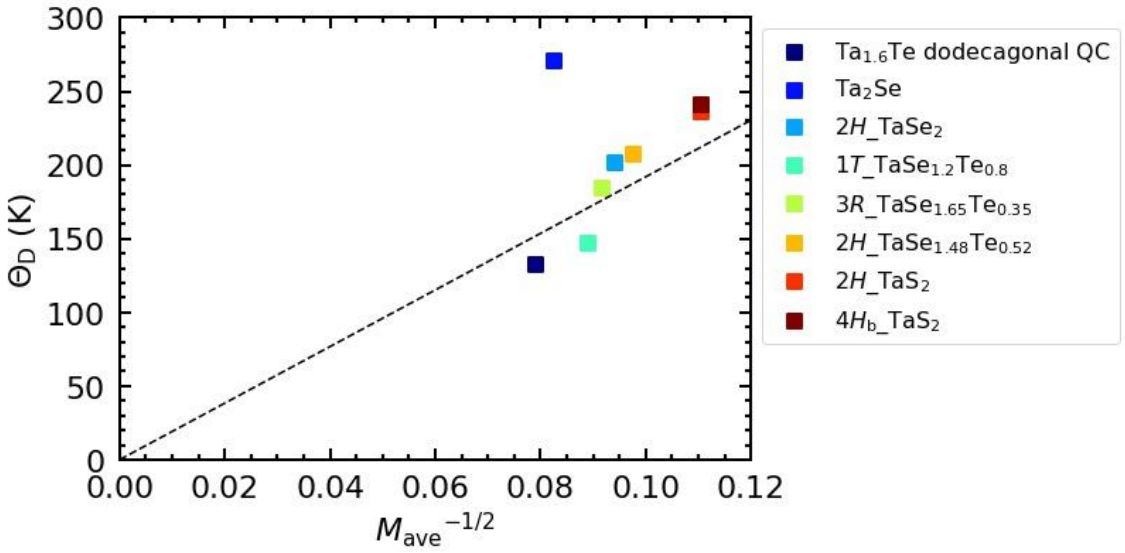

Supplementary Fig. 10 | Debye temperatures (Θ_D) plotted against $M_{ave}{}^{-1/2}$ (M_{ave}: average atomic weight).

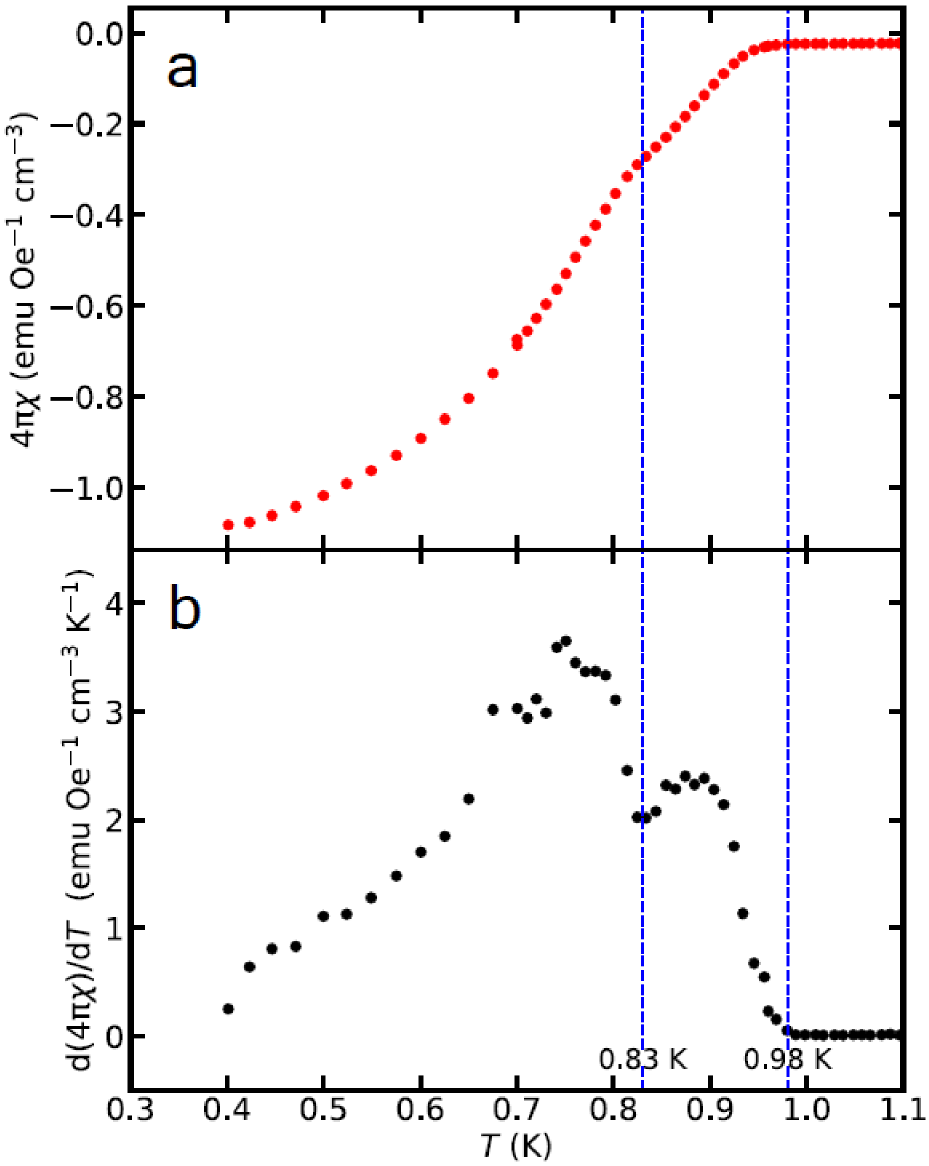

Supplementary Fig. 11 | ZFC magnetic susceptibility data. a, Temperature dependence of the magnetic susceptibility (the same as Fig. 3b); **b**, its temperature derivative. As the temperature decreases, the derivative curve shows a sharp increase at two points: their onset temperatures are 0.98 and 0.83 K.
