# Supplementary material for: Superconductivity in a van der Waals layered quasicrystal
Source: Nat Commun. 2024 Mar 1;15:1529. doi: 10.1038/s41467-024-45952-2 (PMC10907369; doi:10.1038/s41467-024-45952-2)
Supplement: Supplementary file 3 — Description of Additional Supplementary Files [file 41467_2024_45952_MOESM3_ESM.pdf]

Description of Additional Supplementary Files

Supplementary Data 1
Title: Powder XRD data for the $Ta_{97}Te_{60}$ crystal approximant (CA) phase.
Description: Indices, *q*-value, scattering angle 2θ, and relative intensity *I* calculated for the $Ta_{97}Te_{60}$ CA phase using VESTA[Suppl.Ref.4] software with the structural data reported by Harbrecht and Conrad [Suppl.Ref.3]. $q=(4\pi \sin\theta)/\lambda$ and $\lambda=1.5405$ Å.

Supplementary Data 2
Title: Calculated powder XRD data for the dodecagonal quasicrystal (QC) phase.
Description: Indices, *q*-value, scattering angle 2θ, and relative intensity *I* calculated for the dodecagonal QC phase. $q=(4\pi \sin\theta)/\lambda$ and $\lambda=1.5405$ Å.
